# Supplementary material for: Mitigative effect of sodium alginate on streptozotocin (STZ)-induced diabetic neuropathy through regulation of redox status and miR-146a in the rat sciatic nerve
Source: PeerJ. 2025 Mar 24;13:e19046. doi: 10.7717/peerj.19046 (PMC11949120; doi:10.7717/peerj.19046)
Supplement: Supplemental Information 5 [file peerj-13-19046-s005.pdf]

Suppl. Table 1: Primers of qRT-PCR.

	Primers (5′→3′)	Reference number (Vivantis Technologies, Selangor, Malaysia)	Accession number
rno.miR-146a	Forward:<br>GCA GTG AGA ACT GAA TTC CA	1816082875	miRBase MIMAT0000852
	Reverse:<br>GGT CCA GTTT TTT TTT TTT TTT AAC	1816082876	
U6 snRNA	Forward:<br>TTG GAA CGA TAC AGA GAA GATT	1826381783	NR-004394
	Reverse:<br>GGA ACG CTT CAC GAA TTT G	1826381784	
