# Supplementary material for: Mitigative effect of sodium alginate on streptozotocin (STZ)-induced diabetic neuropathy through regulation of redox status and miR-146a in the rat sciatic nerve
Source: PeerJ. 2025 Mar 24;13:e19046. doi: 10.7717/peerj.19046 (PMC11949120; doi:10.7717/peerj.19046)
Supplement: Supplemental Information 6 — Treatment of rats with SA (200 mg/kg b.w.) for 28 days results in reduction of nerve lesions (arrowheads) in diabetic model. Toluidine blue stained sections. Scale bar: 20 µm. [file peerj-13-19046-s006.pdf]

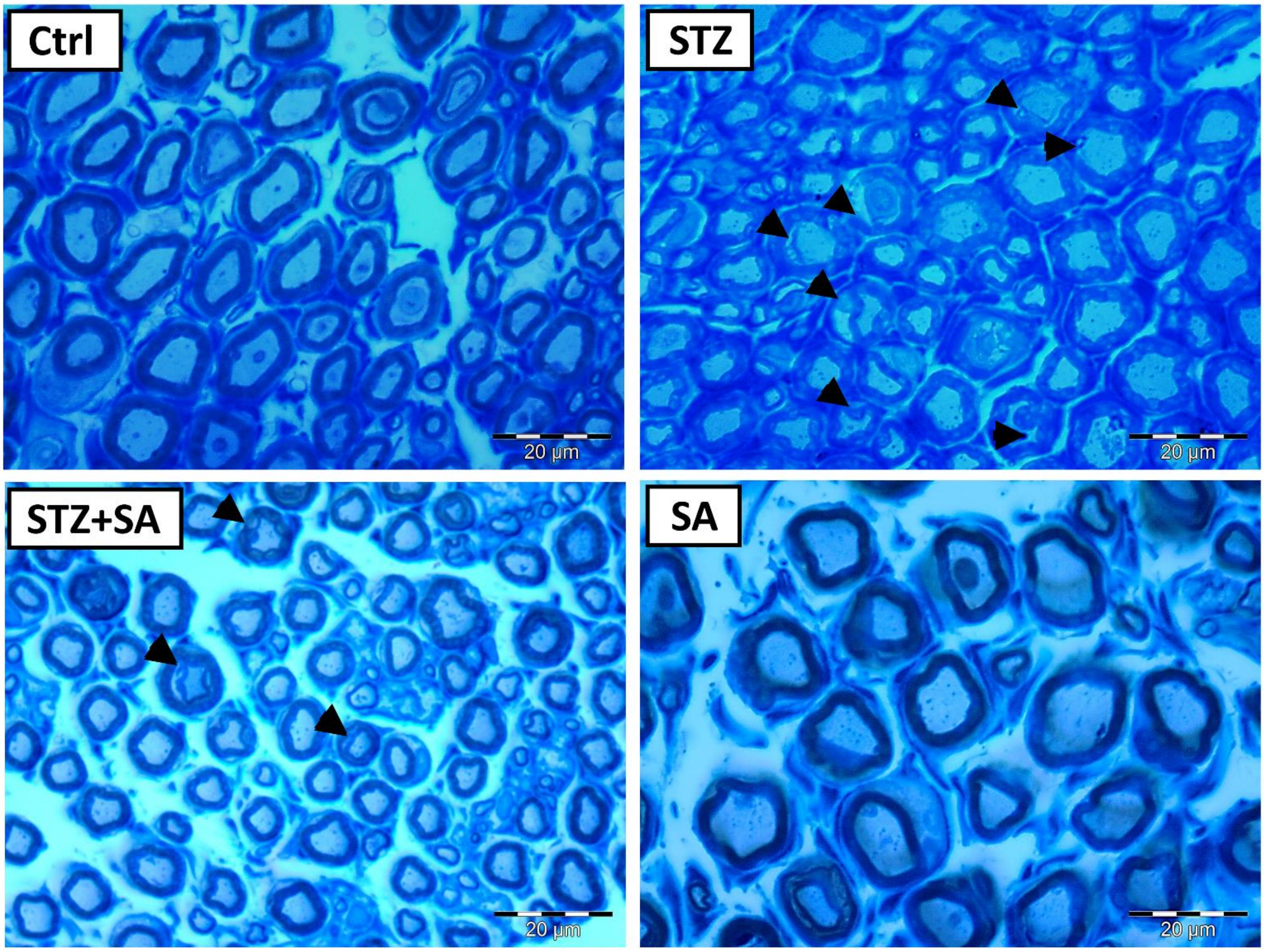

Suppl. Figure 1. Representative photomicrographs of sciatic nerve from different groups, i.e., Ctrl: control, STZ: streptozotocin (diabetic), STZ+SA: streptozotocin+sodium alginate, and SA: sodium alginate). Treatment of rats with SA (200 mg/kg b.w.) for 28 days results in reduction of nerve lesions (arrowheads) in diabetic model. Toluidine blue stained sections. Scale bar: 20 µm.
